# Self-Denial in a Minor Key

**Published:** 1916-05-13

**Authors:** 


### SELF-DENIAL IN A MINOR KEY.

A simple faith in the power of the written word is part of the natural equipment of those who would fain constitute themselves teachers and directors of their neighbours. The quality is an amiable one, and it is hardly for a journalist to deny it. The circumstances of the time have inevitably produced many amateur efforts, and thus the flow of counsel has been abundant. To estimate the results achieved there is no known method, but whatever uncertainty exists on this point it does not seem to paralyse the efforts and the hopefulness of our advisers. Especially abundant have been suggestions for personal and domestic economy, and the very walls carry directions or reproaches to the passing citizen. Of a less official quality are letters to the newspapers, and the favourite fashion is to support these by numerous signatures which presumably are collected for the purpose by the author of the original draft. The hope doubtless is that an attention which might be denied to an individual will probably be granted to a crowd. In this style we are sometimes exhorted and sometimes scolded; our mentors appear at a thousand breakfast-tables—and the world continues its usual way. We ought not to speak unkindly of these efforts. The authors are full of good intentions, as of advice, and fame comes to them but for a passing day.

The latest document of this order is one which urges us to deny our appetite for meat and for alcohol. But the denial is proposed in strictly modest proportions. It is many months since one of the bishops and his academic friends exhorted us to the complete renunciation of alcohol for the period of the war, and another urged the application of similar rigour to tobacco. Now come three bishops who plead but for a single day, and the fragrant weed they leave severely alone. There is no want of precision in their prescription. Monday is for no alcohol, and Thursday for no meat, though on what principle this selection rests is not revealed. In view of the proverbial "dryness" of some of the exercises to which we are accustomed to submit ourselves on the previous day, we are not sure that Monday will be readily welcomed as specially appropriate to the curtailment of beverages, and there is danger of scoffing on this point. It is to be noted, too, that not one of the numerous mentors who sign the letter gives us a guarantee that he has added example to precept. The fact is that suggestions of this order are becoming a little ridiculous, and this latest effort has, we should think, touched bottom. The country has been at war some twenty-one months, the need for economy is evident from the facts, and the proclamation of it has been made by the most authoritative voices. Every citizen who is willing to hear has arranged his own plan of life in accordance with his individual circumstances. And the notion that these suggestions are going to effect any practical good, even when endorsed by bishops and baronets and curates and popular lecturers, is only an evidence of the simplicity and self-satisfaction of its author.

